# Photosensitive drugs: a review on their photoprotection by liposomes and cyclodextrins

**DOI:** 10.1080/10717544.2017.1386733

**Published:** 2017-10-25

**Authors:** Giuseppina Ioele, Michele De Luca, Antonio Garofalo, Gaetano Ragno

**Affiliations:** Department of Pharmacy and Health and Nutrition Sciences, University of Calabria, Rende (CS), Italy

**Keywords:** Photolabile drugs, stability tests, phototoxicity, photoprotection, supramolecular matrices

## Abstract

Nowadays, an exciting challenge in the drug chemistry and technology research is represented by the development of methods aimed to protect molecular integrity and therapeutic activity of drugs from effects of light. The photostability characterization is ruled by ICH (The International Council for Harmonization of Technical Requirements for Pharmaceuticals for Human Use), which releases details throughout basic protocols of stability tests to be performed on new medicinal products for human use. The definition of suitable photoprotective systems is fundamental for pharmaceutical manufacturing and for human healthy as well, since light exposure may affect either drugs or drug formulations giving rise even to allergenic or mutagenic by-products. Here, we summarize and discuss the recent studies on the formulation of photosensitive drugs into supramolecular systems, capable of entrapping the molecules in a hollow of their structure by weak noncovalent interactions and protecting them from light. The best known supramolecular matrices belong to the ‘auto-assembled’ structures, of which liposomes are the most representative, and the ‘host-guest’ systems, of which cyclodextrins represent the most common ‘host’ counterpart. A relevant number of papers concerning the use of both liposomes and cyclodextrins as photoprotection systems for drugs has been published over the last 20 years, demonstrating that this topic captures interest in an increasing number of researchers.

## Introduction

Photostability of drugs has become a very important topic in the field of pharmaceutical research over the last decades. A very limited number of articles on this argument has been published before the 1970 s. A few years later, the United States Pharmacopeia XIX introduced early recommendations toward light-protection of drugs, suggesting the use of simple light-shielding containers as a preventive expedient (The United States Pharmacopeia 19th Rev, 1975). A decisive impulse in this field was generated later by the appearance of several papers dealing with toxic effects to humans caused by some by-products from drug photodegradation. In the last few years, chemical mechanisms and photodegradation kinetics have been described for a more and more increasing number of drugs. Usually, the most common effect of photodegradation on a drug results in the loss or reduction of its pharmacological activity, in some cases accompanied with the formation of toxic by-products. Reviews or collections of monographs that deal very deeply with photolabile drugs and their formulation problems date back to the first decade of the twentieth century (Albini & Fasani, 1998; Piechocki & Thoma, 2006; Tønnesen, 2001, 2004). 

At present, a guide issued by The International Council for Harmonization of Technical Requirements for Pharmaceuticals for Human Use (ICH), adopted in 1996 in Europe and in 1997 in the USA and Japan, prescribes a basic protocol to be applied for the photostability evaluation of new drugs (ICH, 1996). FDA requires such a protocol as a part of tests to be necessarily performed before the marketing of new drugs, for the assessment of drug photostability aimed to exclude unacceptable structure modifications. Impurities coming from both synthesis and degradation must be always identified and quantified. The toxicological aspects of such impurities should be carefully investigated through specific studies, when necessary. ICH photo-stability rules include tests on the pure active principle as well as pharmaceutical formulation and commercial packet. Sunlight irradiation should not be not used during the tests because of its significant variation due to geographical and climatic factors (Moore, 2010).

Two alternative light sources are recommended accordingly: (1) a xenon or metal-halide lamp releasing visible and UV radiations, equivalent to D65 standard for the outdoor daylight and ID65 standard for the indoor indirect light; (2) a fluorescent lamp coupled with an UV 320–400 nm bulb. A careful temperature monitoring is required in order to minimize the interference due to the heat produced by the lamps.

These rules have been subject to criticism and review proposals over the years. In 2010, an important paper by Baertschi and his research team discussed the application of ICH Guideline to drug stability tests (Baertschi, 2010). Later, from 2013 to 2016, the same Authors published a series of three papers for conducting photostability tests on various pharmaceutical forms (Baertschi et al., 2013, 2015). 

In addition to the accurate assessment of the drug’s photostability profile, the development of pharmaceutical formulations that can minimize photodegradation is of paramount importance (Allain et al., 2016). Most therapeutic agents are marketed in solid or liquid formulations. Usually, the solid pharmaceutical forms are more stable to light than solutions.

Strategies aimed at preventing or minimizing drug photodegradation represent an outstanding research topic and new approaches toward the management of this problem are proposed constantly throughout the literature. The most customary method is still represented by the use of containers made with dark or opaque glass, able to halt light of specific wavelengths responsible for photodegradation. This strategy is particularly effective against high-energy radiations, which shows to be the most effective in promoting the transformation of drug compounds. On the other hand, a major drawback is the preclusion of a visual inward inspection.

The formulation employing excipients able to increase photochemical stability of a drug represents an alternative approach widely investigated. Such additives present absorption spectra overlapping those of the drug molecules. Even some antioxidant agents have produced good results in terms of increased photoprotection, due to their ability of preventing free radicals and singlet-oxygen intermediates formation. Ascorbic acid and α-tocopherol are the most studied antioxidants (Ray et al., 2002). In recent years, the use of supramolecular matrices in drug formulations has aroused lively interest since, in addition to the already known properties of increasing the solubility of many substances, they have also been shown to provide valid photoprotection. Supramolecular chemistry is based on two distinct concepts: the self-assembly process, of which liposomes represent the most well-known matrix, and the host-guest inclusion chemistry including cyclodextrins as the most representative example. The host component generally consists of organic molecules forming a central cavity in which the drug, namely the guest, is incorporated. The chemical bonds established throughout the host–guest complexes are necessarily weak, noncovalent bonds.

In this review, the studies dealing the use of liposomes and cyclodextrins (CD) for the drug photoprotection, will be presented. Since these supramolecular systems have also been applied in nonstrict pharmaceutical field but also in related areas such as cosmetics and supplements, their application to some vitamins and sunscreens is also cited in this review, given the high light sensitivity of many of these compounds.

Despite the fact that the study on the supramolecular systems and their application in the pharmaceutical field started many years ago, the review focuses on the latest findings and collects works published over the last twenty years.

## Photoxicity of drugs

Phototoxicity is a skin irritation induced by chemical products (e.g. drugs) which react to light, usually causing more or less intense dermatitis. The phototoxic drugs can be contained in a topical administration or it may reach the skin via systemic circulation following oral or parenteral administration.

Phototoxic reactions occur after the harmful effects of light-activated compounds on cell membranes and, in some cases, DNA. Photoallergic reactions are cell-mediated immune responses to a slightly activated compound. The drugs, or its metabolites, react to light as chromophores and absorb energy, becoming an ‘excited’ state. High-energy content is transferred to the tissues when the molecules return to the fundamental state, causing damage to DNA or cell membranes. In addition to the toxic degradation products, free radicals can also be formed.

It is very important to identify the species responsible for allergy or phototoxic effects. An interesting work of 2007 addresses this issue for dihydropyridines (Pizarro et al., 2007). Some drugs of this class were exposed to stressing artificial light and a significant production of single oxygen, superoxide or both was demonstrated. The formation of these species demonstrates the potential of these drugs to cause phototoxic dermatitis. In particular, felodipine and nimodipine are both capable of generating single oxygen, but exhibiting a different reactivity: the former proves to be very reactive in its ground state while the second in the excited state.

Isotretinoin, deriving from the photolysis of tretinoin, has been proved capable of producing teratogenic effects, so precluding its use during pregnancy (Ioele et al., 2005). The photo-degradation of naproxen leads to toxic products (Isidori et al., 2005). Several sunscreens degrade under sunlight, so reducing their protecting potential against UV rays and giving rise to allergenic derivatives (Perioli et al., 2006).

Endogenous alterations caused by the light can also occur after drug administration for interaction of the drug with endogenous molecules. A well-known example is the irreversible binding of photo-excited psoralens to lymphocytes because of UVA absorption (Song & Tapley, 1979). Two reviews in 1991 and 1997 discuss in depth the phototoxic and phototherapeutic problems of the drugs (Beijersbergen Van Henegouwen, 1991, 1997). Other documents with lots of information on this subject have been published in 2004 and 2008 (Henry et al., 2009; Tønnesen, 2008).

## Supramolecular matrices

Supramolecular chemistry involves only reversible interactions, such as hydrophobic and hydrogen bonds, van der Waals and ion–ion interactions. The number of scientific papers in the supramolecular chemistry field is today impressive and growing day by day up (Atwood & Steed, 2004; Lehn, 2005; Ariga & Kunitake, 2006; Schalley, 2007; Jones, 2008). Over time, supramolecular chemistry has become an important topic in contemporary chemistry, showing remarkable advancements in the areas of chemistry, biochemistry, biology, environmental and materials science.

Molecular self-assembly is a process capable of generating a structural arrangement of particular molecules without any external forces participation. Micellar structures and liposomes represent the most studied self-assembly aggregates. The most interesting property for such matrices is their capacity of dispersing a compound in a certain solvent, in which it is normally insoluble, by incorporation into their nucleus.

**Figure 1.**
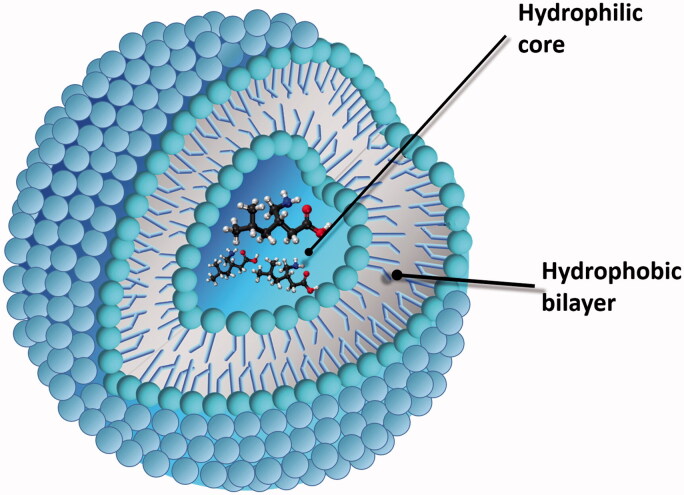
Liposome for drug delivery.

Nowadays, pharmaceutical formulations based on micelles or liposomes are largely used as drug delivery systems (Figure 1). Owing to their small dimension, liposomes can be targeted to highly vascularized zones, such as inflamed or cancerous tissues (Ramamurthy, 2000; Pillai & Panchagnula, 2001; Vincenti & Irico, 2002; Zarif, 2002; Al-Jamal et al., 2005).

**Figure 2.**
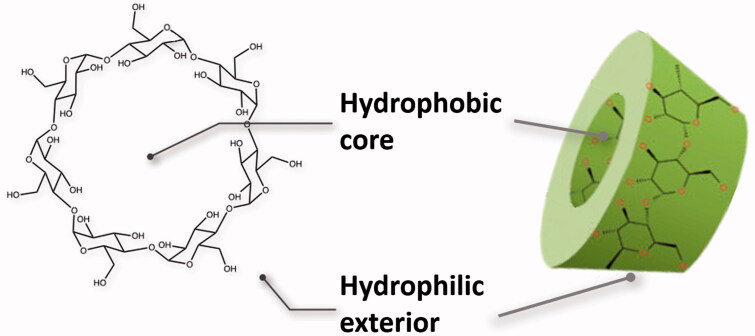
Cyclodextrin: chemical and toroid structures.

CD, but also calixarenes, porfirins and crown ethers, result also of great interest in the host-guest chemistry and are potentially useful for a rationale design of controlled release of drugs (Figure 2), also because the absence of any relevant toxic or antigenic effect (Zhang & Cao, 2001; Yang & De Villiers, 2004). Recent approaches to the controlled release of drugs involve incorporation of the drugs into the matrix of microscopic polymer particles or solid systems as microspheres or microcapsules.

Optimum temperature conditions for the storage of liposomes and CD are below 10 °C. Liposomes stored in buffer at pH 7.4 and ∼4 °C do not show structural changes for 2-3 weeks. After this time, the system destabilizes with the release of free fatty acids generated by lipid hydrolysis. CD solutions can be stored for several weeks at room temperature. However, over 25 °C, the hydrolysis of the structure shows a degradative ring opening.

## Properties and use of liposomes in pharmaceutical field

Liposomes are spherical vesicles formed by phospholipids or amphipathic lipids bilayer. The most used lipids are phosphatidylcholine and phosphatidyl-ethanolamine, made of a long hydrocarbon chain headed with a cation or anionic moiety. Intermolecular forces driving the spontaneous formation of the vesicles in aqueous media are represented by hydrophilic interactions between the ionic ends and hydrophobic interactions. Hydrogen bonds with water molecules participate to the assembly process (Uhumwangho & Okor, 2005). Liposomes are constituted by lipid bilayers, even the multilamellar ones, the overall dimensions varying from 20 nm to several micrometers. The use of liposomal matrices in the pharmaceutical field is well known today. Liposomes are already used for the formulation of drugs (e.g. insulin, antitumor agents) and vaccines or enzymatic preparations.

Hydrophilic drug molecules can be trapped after interaction with the charged ends, while lipophilic drugs are incorporated into the phospholipidic layer or dissolved in the aqueous core (Torchilin & Weissig, 2003; Uhumwangho & Okor, 2005). The success of liposomes for pharmaceutical, but also cosmetic, applications is firstly due to a good bio-compatibility of their components, devoid of any toxic effect, and to their ability in including both lipophilic and hydrophilic substances. Later, liposomes also gained interest for the ability to protect the incorporated substances from chemical and physical agents. Liposomes are today considered as a model for the vehiculation of substances of different nature due to their structural affinity with biological membranes.

The high penetration in the tissues and the controlled release of drugs is another advantageous property for liposomes as a vehicle system (Ahmed & Goldberg, 2004; Immordino et al., 2004; Basu, 2005; Smith, 2005; Uhumwangho & Okor, 2005; Gómez-Hens & Fernández-Romero, 2006; Kassem et al., 2006). Several medical and cosmetic formulations in liposomal matrices are today on the market (Cheng et al., 2004; Rao et al., 2004; Verma et al., 2004). Liposomes have been proposed for topic preparations due to a significantly increase in drug absorption rate (Fang et al., 2006; Ning et al., 2006). Several vaginal formulations are based on the incorporation of drugs into liposomes due to the long lasting release and an improved bioavailability of the drug as well (Ning et al., 2005, 2006, Pavelić et al., 2001, 2005).

Liposomes result of great interest in the anticancer chemotherapy since they can increase selectively the drug release to the cancerous tissue with respect to normal tissues, thus limiting ordinary side effects of the chemotherapy agents (Allen & Martin, 2004; Pan & Lee, 2004; Straubinger et al., 2004; Andresen et al., 2005). Doxorubicin release from light sensitive liposomes proved to be capable of promoting cell death (Yavlovich et al., 2011). Furthermore, a complex liposomal system including doxorubicin and one of its photosensitizer has been assembled in order to treat cancer with magnetic hyperthermia, photodynamic therapy and chemotherapy, at the same time (Shah et al., 2016).

In the modern pharmaceutical industry, niosomes are often used as an alternative to liposomes. Niosomes are nonionic liposomes with dimensions lower than 200 nm formed of amphiphilic nonionic synthetic lipids organized in a bilayer membrane. A preparation of pro-niosomes charged with tretinoin was developed for improving drug efficacy and patient’s compliance, by the reduction of side effects. This new formulation effectively showed an improved efficacy together with a low skin irritation potential when compared to commercially available medicinal products on a number of volunteers (Rahman et al., 2014).

## Liposomes as drug stabilizers

The characteristic function of liposomes to include substances of different nature has led many researchers to study their application as degradation-preventing systems. (Crosasso et al., 2000; Pietzyk & Henschke, 2000; Fatouros & Antimisiaris, 2002). Some reviews summarize the contributions of many authors on the use of liposomes for the light protection of drugs and cosmetics during the last decades of the twentieth century (Bisby et al., 2000; Brisaert et al., 2001; Ragno et al., 1993, 1995, 2006a, 2006b). Subsequently, many other interesting studies and reviews in this field have been published, which we are looking at in this review to report the main ones.

Incorporation into liposomal systems has been deeply investigated for drugs belonging to the class of 1,4-dihydropyridines, known to be photosensitive and leading in most cases to the corresponding pyridine derivatives, devoid of any therapeutic activity (Ioele et al., 2009). In particular, inclusion of amlodipine showed a high protection degree with a rate of degradation comparable to that of solid pharmaceutical formulations. The experiments were monitored by UV spectrophotometry. Liposome and CD complexes showed a degradation of 10%, respectively, at 220 and 480 min, compared with a value of only 10 min for the ethanol solution. Amlodipine pure powder and commercial tablets showed a 10% degradation after 325 and 110 min. (Ragno et al., 2003a).

The previous study was extended to a number of analogs with the aim of developing photostable liquid preparations as an alternative to solid formulations (Ragno et al., 2006b). Analogously to amlodipine, the liposome incorporation of felodipine, nitrendipine, nimodipine and nicardipine showed a satisfactory stability degree. The liposome inclusion allowed a mean drug recovery of 77%, after a light exposure of 30 min of irradiation (21 kJ/min m^2^). Stability proved to proportionally depend on the quantity of incorporated drug. A main drawback encountered in this work was the limited tendency of these drugs to be included into the liposomal cavities. In fact, the presence of bulky functional groups on both rings of the dihydropyridine derivatives made difficult their incorporation, with respect to compounds with low molecular weight and high hydrophilicity. A real benefit from studies on supramolecular complexes could be the preparation of liquid formulations of 1,4-dihydropyiridines, currently formulated only in tablets.

Tretinoin (*all-trans* retinoic acid) is widely used for the treatment of acne vulgaris and others keratin related diseases as well. The drug is used mostly in the acne local therapy with an unavoidable light exposure. Topic applications are, however, limited by the high liability of the molecule to photodegradation, suggesting the development of photo-protected formulations. Tretinoin undergoes photodegradation to the 13-*cis* isomer which in turn is transformed to the 9-*cis* isomer, even though the most important step is the first isomerization of retinoic acid (Brisaert, 2000). The inclusion of tretinoin into liposomes showed a significant increase in light stability, reducing almost completely the isomerization process. Tretinoin showed a residual concentration of 60% after a light irradiance of 3470 kJ/min m^2^, versus a value of 8% of the ethanol solution (Ioele et al., 2005). Photostabilization of tretinoin was also investigated in niosome matrices using a 30 W UV lamp set at 366 nm, showing a protection profile even better than one resulting from liposome inclusion (Manconi et al., 2003, 2006).

Some good results have been also obtained from the development of delivery systems for protein and peptide drugs (Goto et al., 2006). Sericin is a protein largely employed as a nutrient biomaterial for biomedical and cosmeceutic applications, despite its low stability to heat and light. The protein was charged into copolymer-liposomes and stored at different temperatures: 4, 30 and 45 °C. The complex showed high efficiency in the stabilization of structure and concomitantly caused improved dispersibility and biofeasibility (Suktham et al., 2016). Additionally, this formulation proved to be noncytotoxic to human normal cells even at high concentration (10 mg/mL).

Some antibiotic agents have also demonstrated photolability and attempts to minimize degradation have been proposed by incorporation into liposomal systems. Among the antibacterial agents, fluoroquinolones give rise to phototoxic side effects from the formation of reactive oxygen species after UV rays exposure. Photodegradation of ciprofloxacin, ofloxacin and lomefloxacin has been investigated under several UV radiations amounts (Budai et al., 2008). FS-20 lamp (λ = 280–320 nm) was used for irradiation experiments. The UVB-intensity was kept at 1.1 ± 0.1 mW/cm^2^ and the irradiance was in the range 0–22 kJ/min m^2^. Lomefloxacin, indicated by the Authors as the most phototoxic compound, was encapsulated into small mono- and multilamellar liposomes. It was stated that the reduction of the photodegradation process greatly depends on the lipid nature. In particular, unsaturated fatty acid chains in the liposomal bilayer alter the lomefloxacin photodegradation pathway, increasing the probability of CO_2_ loss, frequency of dehydrogenation and then de-fluorination. The main photoproducts were identified by mass spectrometry. Several anticancer drugs are photosensitive. Due to the high toxicity, these drugs and their delivery systems are today widely studied in the pharmaceutical technology field and many of these studies are devoted to the development of photo-protected formulations. The high sensitivity of doxorubicin to natural or artificial light is well known and this represents a relevant drawback since the drug presents a long half-life and accumulates into the derma, where the light exposure is more likely. Doxorubicin encapsulated in polyethyleneglycol-coated liposomes was more stable in comparison to the photodegradation of the free drug and not influenced by concentration. During and after UV exposure, there was no release of the drug from liposomes to the medium. After induced release of the drug, the degradation kinetics of doxorubicin was identical to that of free drug (Bandak et al., 1999).

The main metabolite isolated from Pothomorphe umbellata, namely 4-neroylcatechol, was complexed with liposomes in order to evaluate the drug stability and the cytotoxicity profile. The complex was subjected to forced degradation and monitored by HPLC. The liposome complex resulted able to reduce the degradation rate in all the attempted conditions, showing a half time 15% higher with respect to the pure compound. Interestingly, contrariwise to the behavior of the free drug, the liposome complex showed to be able to protect the erythrocytes from lysis (Gaeti et al., 2014).

Photostability of supramolecular structures charged with *(E)-*resveratrol, together with the skin penetration profile of nanostructured formulations, was investigated. Several supramolecular structures such as liposomes, lipid-core polymeric nanocapsules, nanospheres and solid lipid nanoparticles, were designed as carriers for this nutraceutical compound. Liposomes resulted as the most effective matrix for long preserving resveratrol concentration. On the other hand, the small size complexes showed a reduced physical stability under UVA radiations (Detoni et al., 2012). New and more thorough studies are today needed to optimize the vehiculation in liposomes of resveratrol, but also of other nutraceuticals, also because of the increasing importance that these substances are taking for the human health.

The effect of lipid microparticles carrier systems on the light-induced degradation of melatonin was also investigated. Photodegradation experiments were carried out by using a Xenon lamp, wavelengths under 290 nm were filtered and temperature was maintained under 30 °C. The solar simulator emission was maintained at 500 W/m^2^. Photolysis experiments demonstrated that melatonin photo-decomposition markedly decreased after tristearin and phosphatidylcholine-based liposphere encapsulation (the degradation rates were 19.6% and 5.6% for free and incorporated melatonin, respectively) (Tursilli et al., 2006).

Since the proneness to the degradation of many vitamins has emerged, even under light exposure, the attention of many researchers has been directed to the use of supramolecular systems to define protective systems. It is well known that vitamin E (α-tocopherol) is photolabile and several attempts have been made in order to define photo-protective formulations by liposome vesicles incorporation. The best results was obtained by dispersion of the vitamin in the inner phase in the presence of the antioxidant ascorbic acid in the outer phase. No detectable alteration of the molecule was recorded up after a nine hours irradiation time under UVB lamp in a range of 290–320 nm (Gallarate et al., 2004).

The use of liposomes to incorporate sunscreens has also yielded effective results. The encapsulation of the sunscreen avobenzone into liposomes using isolecithines resulted highly efficient. A degradation of 22.07% against 32.96% of nonencapsulated avobenzene was recorded when subjected to irradiation in a solar simulation chamber at a power of 1000 W/m^2^ for one hour (Madrid & Cabrera, 2011).

In using the niosomes as an alternative to the liposomes, an interesting approach is the use of niosome vesicles made of nonionic surfactant, developed by Abdelkader et al. (2012) to attempt autoxidation prevention of naltrexone hydrochloride. This drug is a promising agent for the treatment of cornea diseases associated with diabetes mellitus (diabetic keratopathy). Unfortunately, naltrexone suffers from stability problems due to autoxidation reactions. An aqueous solution of the drug was maintained under exposure to artificial daylight (10.000 lux) causing a remarkable drug degradation. The niosome formulations were shown to be able to significantly (*p* < .05) protect the incorporated drug from photo-promoted oxidation. A 1–3-fold decrease was estimated for the drug encapsulated in niosomes compared with the drug solution.

In the last few years, the attention of many researchers has been attracted by the inclusion of the binary complex CD/drug into liposomes. The effect coming from the two matrices combination seems able to offer effective drug protection against several degradation agents. Photostability of barnidipine in combined CD/liposomes was investigated with the aim to prepare liquid formulations of this drug, as an alternative to the solid commercial specialties (Ioele et al., 2014). The residual percentage of barnidipine in liquid preparations of liposomes and CD was 42.90 and 72.03%, respectively, compared with a value of 29.81% for the drug in ethanol, after a radiation exposure of 225 kJ/minm^2^. When the drug–CD complex was in turn entrapped in liposomes, a residual percentage of 90.78% was reached, very close to the value of 96.03% carried out in degradation of tablet formulations.

Incorporation of the hydroxypropyl/CD complex with the sunscreen butyl methoxydibezoylmethane into lipospheres was studied in order to assess the effectiveness of this system on drug photostability. The incorporation ternary system proved to be more effective than the binary complex or the simple drug/liposphere complex in improving photostability of the drug. The samples were irradiated by a solar simulator equipped with a Xenon lamp, an optical filter to cutoff wavelengths shorter than 290 nm and an IR-block filter to avoid thermal effects. The solar simulator emission was maintained at 500 W/m^2^ (Scalia et al., 2006).

A drug-in-CD-in-liposomes was applied as a carrier system for anethole, an essential oil component. The free compound in aqueous solution was found to be unstable upon exposure to UV irradiation. Under irradiation, anethole disappeared completely after 2 h. Hydroxypropyl-β-CD/anethole inclusion complexes were encapsulated into liposomes and exposed to UV light. Compared to the aqueous solution, a protection factor of 66.7 times was obtained (Gharib et al., 2017).

Due to their lipid-based structure, liposomes themselves showed to be in turn sensitive to light (Bisby et al., 2000). Therefore, a careful monitoring of the photostability of liposomal matrices is always required. Since degradation is promoted by both temperature and light exposure, low temperature and light shielding are required during liposomes manipulation and storage. The addition of anti-oxidant agents could be desirable to optimize the stability of preparations.

Table 1 summarizes the literature meaning on the photostabilization approaches by incorporation in liposomes.

**Table 1.** Papers concerning the photostabilization of drugs by liposome incorporation.

Drug	Photostabilization in liposomes
1,4-Dihydropyridines	Ioele et al., 2009; Ragno et al., 2003a, 2006b
4-Nerolidylcatechol	Gaeti et al., 2014
alpha-Tocoferol	Gallarate et al., 2004
Amlodipine	Ragno et al., 2003a
Anethole	Gharib et al., 2017
Avobenzone	Madrid & Cabrera, 2011
Barnidipine	Ioele et al., 2014
Butyl methoxydibenzoylmethane	Scalia et al.,^2006^
Doxorubicin	Bandak et al., 1999
Fluoroquinolones	Budai et al., 2008
Melatonin	Tursilli et al., 2006
Naltrexone	Abdelkader et al. 2012
Penicillins and cephalosporins	Uhumwangho & Okor, 2005
Resveratrol	Detoni et al., 2012
Sericin	Suktham et al., 2016
Tretinoin	Brisaert, 2000; Brisaert et al., 2001; Ioele et al., 2005 Manconi et al., 2003; Manconi et al., 2006

## Characteristics and use of cyclodextrins for pharmaceutical applications

CD are known since 1953, but their wide application in pharmaceutical formulations was precluded by the high production costs. During the 1970 s, the production was greatly implemented due to the progress of biotechnology, which led to a significant cost cutting. Studies on the utilization of CD in the formulation of supramolecular matrices rose in turn up. Today, CD are produced at a reasonable cost directly from starch under the action of CD glycosyltransferase (CGTase), an enzyme easily accessible from various cultures of bacteria. A storage temperature of 2–8 °C is required.

CD are characterized by a high inclusion potential toward many compounds and the absence of evident toxic effects as well. The functional molecules are complexed within a hollow in the oligomer structure, wherein they are safely stored and protected from air and external oxidation agents. For all these reasons, CD have now become the most important inclusion system in pharmaceutical field as well as in food and cosmetic manufacturing. CD and several derivatives are studied as pharmaceutical excipients to improve water solubility, stability and bioavailability.

From a structural point of view, CD are cyclic oligosaccharides formed of glucopyranose monomers linked by α-(1,4)-glyosidic bonds. Based on the number of monomers (6, 7 or 8), they are called α, β or γ CD. The spatial arrangement of CD defines a sort of frustum of cone rigid shape with an inner hollow capable to accommodate a variety of functional substances. The structure presents a hydrophilic outer surface while the inner surface of cavity is almost hydrophobic. Similarly to liposomes, the complexes of CD/compounds are based on reversible interactions such as hydrogen bonds and London dispersion forces.

Some recent reviews are particularly rich in information about CD and their application in the pharmaceutical field (Cagno, 2017; Iacovino et al., 2017).

The most common application of CD in the pharmaceutical field is toward the increase of drug solubility in aqueous media. However, a remarkable role for CD refers to their capacity to protect drugs from different degradation processes. In particular, CD are studied for their potential of stabilizing several reactive cytotoxic drugs (Chakraborty & Naik, 2003). A number of ocular formulations including such inclusion systems are already on the market (Sigurdsson et al., 2005). Moreover, administration of drugs by inhalation could take advantage by CD vehiculation, leading to an improved absorption of various steroids, peptides and proteins (Gu et al., 2005; Arima et al., 2017; Wei, 2017).

## Cyclodextrins as drugs stabilizers

The potential of CD in improving the stability of drugs has been widely investigated in pharmaceutical technology. A number of molecules entrapped in CD matrices have shown an improved stability under various stressing conditions of light, heating, oxidation agents, moisture. Many citations regarding the ability of these matrices to protect the drugs from light are also provided. Some reviews published some years ago provide very detailed information on the use of CD in drug delivery and especially for topical formulations. Many references regarding the ability of these matrices to protect the drugs from light are also provided. (Cal & Centkowska, 2008; Challa et al., 2005). Usually, the drug/CD ratio is 1:1 and the complex stability is generally higher at low temperature (Loftsson et al., 2001; Valle, 2004).

Similarly to the liposomal systems, photodegradation of 1,4-dihydropyridine antihypertensive agents, very sensitive to light, can be greatly lowered by CD incorporation, as reported by several recent studies herein reported. Nifedipine, the lead compound of this series, proved to noteworthy gain stability after complexation with hydroxypropyl-CD and dimethyl-CD, even in solid state (Bayomi et al., 2002). Photochemical degradation of a series of 1,4-dihydropyrine derivatives was investigated by comparing the free compounds with their corresponding CD complexes. The samples were exposed to UV radiation by a Fluotest lamp, model NN 15/30, λ = 254 nm from a 30 cm distance. Photodegradation was found to be dependent on the position of the NO_2_ group in the phenyl ring. Degradation rate of the *ortho*-isomer in the CD complex was 200 times slower than that for this compound in the solid phase. In contrast, the presence of halogen groups caused a 4-fold increase in the photodegradation rate (Mielcarek, 1997). Photochemical transformation of manidipine leads to both nitro-phenylpyridine and nitrous-phenylpyridine derivatives. In this case, CD complexation reduced the photodegradation rate, although to a lesser extent with respect to analogs belonging to the same series (Mielcarek & Szamburska, 2005). Photostability of amlodipine was also checked in CD as well in liposomes and microspheres (Ragno et al., 2003a). The best results were obtained by CD inclusion complex, which showed a 90% residual degradation after nine hours of light exposure.

A comparative study on the effects of photoprotection by supramolecular systems on eleven 1,4-dihydropyridine derivatives was also performed (Ragno et al., 2003b). Lacidipine, manidipine, nifedipine and nimodipine resulted the more sensitive molecules, as they degraded less than 50% after 5 minutes, in aqueous solution. The best results in photostabilization were obtained for amlodipine, felodipine, nisoldipine and nitrendipine complexed with CD. A mean drug recovery of 90%, after 30 min of light exposure with an intensity of 21 kJ/min m^2^was carried out from CD inclusion complexes. It was also established that steric factors greatly influence the drug incorporation rate into CD and therefore the photostabilization grade. In fact, lercanidipine and manidipine, both bearing bulky substituents, showed a low drug inclusion value with a consequent less marked increase of photostabilization.

Photostability studies of nicardipine were performed by inclusion complexes with various CD and exposed to UVA–UVB radiations (Xe-arc lamp) for increasing irradiation times. The photodegradation process was monitored by capillary electrophoresis, able of achieving enantio-resolution of the racemic form (Pomponio et al., 2004). β-CD, hydroxypropyl-α-CD and (2-hydroxyethyl)-α-CD showed a photoprotective effect, while γ-CD, methyl-β-CD, hydroxypropyl-β-CD, hydroxypropyl-CD and γ-CD did not promote any increase in photostability. Stereoselective photodegradation of *rac*-nicardipine was observed for the β-CD complex and two distinct photodegradation profiles, with different kinetic constants, were observed for the two enantiomers. These results are very interesting because suggest, in any case, the possibility of different photodegradation profiles for the enantiomers of a chiral photoreactive drug.

Sublingual tablets of isradipine and β-CD were developed in order to optimize administration of this drug. Photostability studies were conducted by differential scanning calorimetry and X-ray diffraction. The complexed drug was more stable if compared to the pure drug and the dispersibility was also improved (Himabindu et al., 2013). In another paper, tablets prepared by direct compression of the CD complex with hydroxypropylmethylcellulose resulted more stable than the pure drug after 4 days irradiation (Park et al., 2013). Improvement of photostability of clinidipine was also attempted. The drug was mixed with hydroxyl-β-propyl CD and the results obtained after light exposure (4500 lx at 40 °C) proved an increased stability for the complexed drug while the dispersibility increased by 10,000-fold (Hu et al., 2012).

Tretinoin is a very photosensitive compound, as above described (Brisaert, 2000). CD showed a good protective action for this drug, with only a minimal degradation after 48 h in phosphate buffer, pH 7.4 (Semenova et al., 2002). In a subsequent study, tretinoin samples, in incubator at 37 °C, 30 cm from three 6 W fluorescent tubes, showed a decrease to the 20% of its initial drug concentration in 30 min, leading to a number of by-isomers, some of them being toxic. Complexation with α-CD and hydroxypropyl-β-CD resulted in a stability increase, while incorporation into β-CD did not produce any significant photoprotective effect (Yap et al., 2005). In this study, molecular modeling was adopted to illustrate the complexation structures, proposing the computational molecular modeling as a valid approach to predict binding and protecting ability of various CD.

In another paper, stability of free tretinoin in methanol and the complexed drug with β-CD was compared (Caddeo et al., 2007). The drug/CD complex showed a clear increase in drug stability, under exposure to both UV and fluorescence light. The amount of intact drug was 20–25% in complex form after 30 days under light, compared to the complete degradation of the free form after the same time.

CD proved to be ideal vehicle agents also for anti-inflammatory preparations. Photolability of naproxen in aqueous solution, exposed under six 40 W fluorescence lamps, was significantly reduced by addition of polyvinylpyrrolidone to the complex of the drug with hydroxypropyl-β-CD (Valero & Esteban, 2004). Complexation of diflunisal, which is known to induce phototoxicity, with CD showed a high rate of photostabilization of the drug (Sortino et al., 2003). Complexes of sulfanilamide with β-CD and hydroxypropyl-β-CD were prepared and subdued to stressing light. The latter complex proved to be more photo-stable than the pure drug and the β-CD complex as well (Tačić et al., 2014). Sodium diclofenac was complexed with hydroxypropyl-β-CD and the subsequent physic-chemical characterization was performed in order to evaluate drug photo-stability. The results demonstrated that the photochemical degradation rate of the complexed drug was significantly reduced when an optimal 4:1 host:guest ratio was used (Manikandan et al., 2016). Rhein, a major metabolite of the prodrug diacerein is used in the treatment of osteoarthritis and diabetic nephropathy. Its incorporation in CD in a 1:1 host-guest system seems to efficiously improve both dispersibility and stability when exposed to the visible light of a lamp (800 lx) (Petralito et al., 2009).

Photodegradation is also considered important for the antihistaminic drugs, which are often administered topically and therefore more exposed to effects of light. The effect of β-CD complexation on the photostability of antazoline, xylometazoline and nafazoline was studied. Molecular mechanics allowed to set a 1:1 ratio structure for the inclusion complexes, which in turn gave an idea about the energetically preferential structure of the complexes and consequent increase in stability (Bani-Yaseen et al., 2009). A significant reduction of photodecomposition was also obtained after complexation of triprolidin (Ndlebe et al., 2004).

Several sunscreens complexed with β-CD have been tested to verify possible photostability increases. The complex interaction of the sunscreen 4-methylbenzylidene camphor with α-hydrophylic-CD, β-CD and γ-CD in aqueous solution was studied by exposition in a solar simulator equipped with a Xenon lamp, an optical filter to cut off wavelengths shorter than 290 nm and an IR-block filter to avoid thermal effects. Irradiation was fixed at 750 W/m^2^. The light-promoted degradation markedly decreased for the β-CD complex with a rate of 7.1% for the complex versus 21.1% for the free drug, as calculated by HPLC (Scalia et al., 2007). Flavonoids are a well-known class of natural pigments playing a protective role in plants toward low wavelengths. This property is considered potentially useful for the protection of biological targets such as lipids, proteins and coenzymatic factors. The most representative members of this class, showing a 3-OH-flavonoid structure, present significant photoreactivity (Smith et al., 2000). Pure flavonoid molecules and CD-encapsulated version were exposed to UV-A radiation in a chamber emitting in the range 310–390 nm. Once again, the inclusion process showed a ca. threefold reduction of the photodegradation quantum yield (Tommasini et al., 2004).

The photoprotective ability of CD has been also tested on chemotherapeutic drugs. The effect on oxolinic acid, a bactericidal chemotherapeutic belonging to the quinolone family, effective in the treatment of acute and chronic infections of the urinary tract, was checked by complexation with hydroxypropyl-β-CD. The careful examination of photodegradation profile confirmed a first-order kinetics reaching photostabilization peak up to 94% (Orfanou et al., 2009). Ofloxacin, an effective fluoroquinolone derivative, which suffers from a marked photochemical sensitivity, was complexed with β-CD. An increase in solubility of the drug was the unique result obtained, since any reduction of unstability of the drug was not detected in this case (Pandya et al., 2010). Effects of encapsulation in CD on solubility, photostability and antifungal activities of some phenylpropanoids were investigated. Photodegradation experiments were carried out by using 10 UVC lamps (254 nm, 15 W) and a Multirays apparatus. Results showed that encapsulation in CD significantly increased dispersibility and photostability of the studied drugs (from 2- to 17-fold and 2- to 44-fold, respectively). Encapsulation of phenylpropanoids, despite a reduced antifungal activity, could be helpful to solve drawbacks such as solubility and stability (Kfoury et al., 2016).

Some antireflux drugs also show degradation under light. Lansoprazole, a proton pump inhibitor, was studied after complexation with β-CD and 2-hydroxypropyl-β-CD. The drug solubilization rate and stability significantly improved in both complex preparations. The first complex resulted more stable under lightening than the corresponding β-CD one, due to the inclusion of the sulfonyl moiety into the CD cavity (Lu et al., 2012). The photo-oxidation process of ranitidine in solid state, irradiated for 48 h in a stressing light cabinet, leads to variation of its color and unpleasant odor as well. The inclusion in a CD complex largely prevents transformation, probably due to the inclusion of the sensitive furan moiety of the molecule into the CD cavity (Jamrógiewicz et al., 2014).

Other photosensitive drugs, belonging to different therapeutic classes, have been incorporated into CD to minimize degradation. The inclusion complex of β-CD with methotrexate has been prepared with the aim to improve drug water solubility and, at the same time, decrease drug photosensitivity. Light decomposition markedly decreases after complexation (Bourkaib et al., 2013).

Photostability of ascorbic acid was studied by comparison of a ternary triethanolamine and β-CD complex and a quaternary complex obtained by further addition of hydroxypropyl-β-CD under artificial and diffused light. Sample solutions were irradiated with a Philips mercury arc lamp (range 312–577 nm) transmitting light corresponding to exposure behind a glass window. Another set was positioned under daylight fluorescent tubes (Philips, TLT 40 W/54, range 400–600 nm). The obtained data showed that these complexes strongly reduce the drug photodegradation in a range of 11- to 35-fold, depending on the ligand concentration and the irradiation source (Garnero & Longhi, 2010).

Quercetin is a flavonoid endowed with a strong antioxidant activity and it is present in a variety of vegetables and fruits. Its application in nutraceutical products is, however, limited by low water solubility and scanty stability. In a recent study, both dispersibility and stability of quercetin were improved by complexation into nano-sponges CD based. Five different types of nano-sponges were assembled by reacting varying amounts of diphenylcarbonate with β-CD. Dissolution of quercetin-containing nano-sponges was significantly greater than the free drug itself and a remarkable increase of photostability was also achieved (Anandam & Selvamuthukumar, 2014). The antibiotic doxicyclin is a drug highly sensitive to light, so limiting the stability of its pharmaceutical formulations. Once more, the inclusion of the drug into CD gave rise to a complex characterized by a higher drug stability in aqueous solution (Kogawa et al., 2014; Savic-Gajic et al., 2016).

The most important studies regarding the incorporation of drugs in CD matrices for stabilization aims are summarized in Table 2.

**Table 2.** Papers concerning the photostabilization of drugs by cyclodextrin incorporation.

Drug	Photostabilization in cyclodextrins
1,4-Dihydropyridines	Mielcarek, 1997; Ragno et al., 2006b
13-cis-Retinoic acid	Yap et al., 2005
3-Hydroxyflavone	Tommasini et al., 2004
4-Methylbenzylidene camphor	Scalia et al., 2007
5-Hydroxyflavones, flavonols	Smith et al., 2000
Amlodipine	Ragno et al., 2003a
Antazoline, Xylometazoline, Nafazoline	Bani-Yaseen et al., 2009
Ascorbic acid	Garnero & Longhi, 2010
Carvedilol	Savic-Gajic et al., 2016
Cilnidipine	Hu et al., 2012
Diclofenac	Manikandan et al., 2016
Diflunisal	Sortino et al., 2003
Doxycycline	Kogawa et al., 2014; Savic-Gajic et al., 2016
Isradipine	Himabindu et al., 2013; Park et al., 2013
Lansoprazole	Lu et al., 2012
Manidipine	Mielcarek & Szamburska, 2005
Methotrexate	Bourkaib et al., 2013
Naproxen	Valero & Esteban, 2004
Nicardipine	Pomponio et al., 2004
Nifedipine	Bayomi et al., 2002
Ofloxacin	Pandya et al., 2010
Oxolinic acid	Orfanou et al., 2009
Phenylpropanoids	Kfoury et al., 2016
Quercetin	Anandam & Selvamuthukumar, 2014
Ranitidine	Jamrógiewicz et al., 2014
Retinol	Semenova et al., 2002
Rhein	Petralito et al., 2009
Sulfanilamide	Tačić et al., 2014
Tretinoin	Caddeo et al., 2007
Triprolidine	Ndlebe et al., 2004

## Conclusions and perspectives

Studies on drug degradation are receiving great attention from the scientific community. One of the main impulses in this research field lies in the toxicity acquired by some drugs after exposure to light. Drug-induced photosensitivity is obviously more commonly found for topical applications, often causing skin irritations, but also oral or parenterally administered drugs can induce phototoxicity. Generally, the amount of drug needed to cause photoallergic reactions is considerably lower than that required for phototoxic reactions.

In order to avoid such risks, proposals are being multiplied to provide maximum photoprotection to drugs and thus minimize the effects of light on the integrity of products and their therapeutic activity. The definition of photoprotective systems seems to be of paramount importance in the modern pharmaceutical industry. Recent studies are focusing on new formulations that incorporate the drug into macromolecules that can exert physical light protection. Among them, supramolecular systems are characterized by trapping the drug into a cavity of their structure, involving only weak binding interactions.

Liposomes and cyclodextrins are the supramolecular systems that have been most successful in pharmaceutical technology for the last 20 years and still promise to achieve new results.

Although liposomes have so far been of great interest for topical preparations, some difficulties have been identified in the penetration of drugs through the skin. In addition, in some cases, liposomes are unable to penetrate the deep layers of the epidermis. Recent approaches suggest the design of flexible liposomes, based on phospholipids in the presence of ‘activator’ molecules (for example, surfactants that destabilize lipid bilayers of vesicles and increase deformability of the system). These systems have been shown to improve skin diffusion of proteins and peptides such as insulin, vaccines, nonsteroidal anti-inflammatory drugs such as ibuprofen and diclofenac (Liu et al., 2013). Recently, very important approaches show the development of temperature-sensitive liposomes that allow the release of anticancer drugs directly into neoplastic tissues as a result of a higher temperature of these tissues (Park et al., 2013).

A proper and beneficial use of cyclodextrins in the pharmaceutical field requires careful evaluation of a number of technological and biological parameters. The choice of CD can affect the performance of drug/CD complex. It is necessary to take into account the dimensions of the CD cavity and the drug to be incorporate, the charge of the drug, which can be influenced by the pH of the environment and varying the strength of the complex.

The supramolecular systems that are generating great interest in the pharmaceutical field are based on drug–cyclodextrin complex incorporated in turn in the aqueous core of liposomes, by defining the so-called drug-in-CD-in-liposomes systems (Hatzi et al., 2006; Piel et al., 2006; Salem & Düzgünes, 2003). The use of these matrices could be particularly advantageous for photosensitive drugs that achieve greater protection from light.

Supramolecular system-based drug delivery systems are proving to overcome the limitation of conventional drug delivery systems, mainly characterized as controlled release systems and targeted drug delivery systems. The main objective is to obtain systems with good loading and controlled release of drugs, at the same time characterized by low toxicity.

In any case, the studies on photostability and the application of photostability tests conducted under international guidelines remain an important part of the formulation studies, enabling the development of stable, safe and effective products.
